# Efficiency in First-Aid Work

**Published:** 1913-09-20

**Authors:** 


*To the Editor of* The Hospital.

### Efficiency in First-Aid Work.

*To the Editor of* The Hospital.

Sir,—It is apparent to any keen observer that there are thousands of persons yearly taking a course of perhaps half a dozen lectures on first aid, and obtaining a certificate, but the question that must arise is—What efficient service do these individuals render the public at large? It is perfectly true that among the voluntary-aid detachments and ambulance brigades, etc., there is a percentage of members whose knowledge is such as to be of real value, but it only needs observation to see the inefficiency of the majority of first-aid certificate holders. How many such know the antidotes for the poisons? How many really know how to apply pressure at the arterial points or could differentiate between the variety of fits? Such persons are not beneficial to the public; their little knowledge is of the greatest danger. What, then, I ask, is the remedy? And at once a danger arises—the "first-aider" must never be allowed to consider himself "trained" in the professional sense of the word or presume to take upon himself the *rôle* of medical man or hospital nurse—but what is wanted is not to instruct thousands annually by a few lectures and grant certificates wholesale, but to narrow down the openings for such students by a thorough and lengthy course of teaching with practical demonstration—it might be, if circumstances permitted, in the casualty departments of our great hospitals—to be concluded with an examination that would severely test the capabilities of the candidate.

It is, of course, not suggested that teaching of hygiene, elementary first aid, or home nursing should be banned altogether, but rather that certificates should be issued, after thorough instruction and examination, to a smaller number of really efficient persons. Such a course would have the result of weeding out those whose interest is merely passing, but would retain those who are in real earnest and realise what is expected of them, and while doing all in their power to make themselves proficient in their work would never presume to take upon themselves the professional *rôle*.—Yours faithfully,

Ex-Hospital Chaplain.

